# Experimental crossbreeding reveals strain-specific variation in mortality, growth and personality in the brown trout (*Salmo trutta*)

**DOI:** 10.1038/s41598-018-35794-6

**Published:** 2019-02-26

**Authors:** Anni Ågren, Anssi Vainikka, Matti Janhunen, Pekka Hyvärinen, Jorma Piironen, Raine Kortet

**Affiliations:** 1 Department of Environmental and Biological Sciences, University of Eastern Finland, P.O. Box 111, FI-80101 Joensuu, Finland; 2 Natural Resources Institute Finland (Luke), Aquatic production systems, Myllytie 1, FI-31600 Jokioinen, Finland; 3 Natural Resources Institute Finland (Luke), Aquatic population dynamics, Manamansalontie 90, FI-88300 Paltamo, Finland; 4 Natural Resources Institute Finland (Luke), Aquatic population dynamics, Yliopistonkatu 6, FI-80100 Joensuu, Finland

## Abstract

Although hybridization between populations with low genetic diversity may induce heterosis, it can also lead to reduced fitness of hybrid offspring through outbreeding depression and loss of local adaptations. Using a half-sib mating design, we studied on brown trout (*Salmo trutta*) how hybridization of migratory hatchery-strain females with males from various strains would affect early mortality, growth and personality in F_1_ offspring. No differences in mortality or alevin body length were found between the crossing groups by the end of the yolk-sac stage. At later developmental stages, higher mortality and slower growth in one of the geographically distant hybrid groups indicated potential outbreeding depression. The personality component indicating boldness and exploration tendency showed fairly low genetic variation and no phenotypic differences among the crossing groups while the personality component related to freezing behavior indicated stronger freezing responses in the purebred and local cross strain when compared to the two other strains. However, the purebred hatchery strain possessed stronger additive genetic tendency for boldness and explorative behavior, and weaker genetic tendency for freezing behavior, when compared to the wild × hatchery hybrid group. Our results add to the cumulating evidence of risks related to the stocking of fish strains from non-native origins.

## Introduction

Due to large-scale intentional releases of hatchery-reared fish and unintentional escapes of domesticated fish from aquaculture facilities, many of the world’s wild salmonid populations are under the threat of introgression by domesticated and non-native conspecifics^1–3^. Although hybridization between severely inbred populations may induce heterosis, also known as hybrid vigor^4^, it can also lead to reduced fitness and even outbreeding depression of the hybrid offspring when compared to wild, locally adapted, individuals^5–7^. Hatchery-rearing typically occurs in purpose of supplementing the wild fish stocks with additional fish that should resemble the wild fish (fish born in the wild environment), while in fish farming domestication occurs in response to selection for better growth performance and the fish enter the environment only accidentally. In natural conditions, survival and reproduction rates of both hatchery-reared and farmed fish are usually considerably lower than those of wild individuals^8–11^. Although experiments on intraspesific farmed-wild hybrids have yielded mixed results, particularly studies on farmed vs. wild Atlantic salmon have shown clear negative effects of hybridization on the survival of fish in the wild^6,12^.

In addition to possible differences in survival, hatchery/farmed-wild hybrids have been shown to differ from their wild parental strain in several behavioral traits, including aggressiveness^5,13^, risk-taking^5,14^ and susceptibility to angling^15^. However, not all the studies have documented differences between farmed-wild hybrids^16^. Moreover, a comprehensive analysis of possible differences in personality (behaviors that vary predictably among individuals, and are consistent across time and/or contexts within individuals)^17^ between purebred strains and hybrid strains is yet lacking. Nevertheless, the reduced fitness of hybrid fish in comparison to native fish is potentially partially explained by non-optimal or inappropriate behavioral responses in a given environment^8^. This behavioral effect of introgression may be significant for salmonids, since local adaptations are common and the “optimal behavior” can vary greatly between populations^8,18^. Furthermore, according to studies including F_2_ and wild backcross hybrids, the effects of hybridization on fitness and/or behavior are not limited to first generation wild-hatchery hybrids but can be carried on to the following generations^6,7,19,20^. Different natural populations or even distinct species can sometimes hybridize also in the wild with unwanted effects on the conservation of genetic integrity and diversity^21^. While any introduction of a remote population of the same species should be considered as an issue for salmonid management and conservation, sometimes releases of hatchery-reared fish are a necessity as the original stocks have completely disappeared or only small-sized resident fish with minor fisheries importance are found^22^.

There is ample evidence that unintended hatchery-induced selection and relaxation of natural selection pressure can rapidly cause both genotypic and phenotypic changes in hatchery broodstocks^23,24^. Considerable genetic changes can occur even during a single hatchery generation^25,26^. General examples of traits influenced by unintended domestication include growth^27^ and age at maturation^28,29^, but different aspects of behavior are also affected by domestication^27,29,30^. In general, hatchery fish are often, but not always, more aggressive than wild fish^31^. Hatchery fish are also more insensitive to predation risk^14^ and possibly more resilient to stress^32^. In many features, farmed-wild hybrid fish often seem to either resemble their domesticated parent strains or be intermediate compared to pure wild and purebred hatchery individuals^5^.

Another threat posed by stockings is the genetic erosion, where the limited gene pool shrinks even more when reproductive individuals die off before reproducing due to competition, or the scenario where between-population genetic diversity diminishes due to regional genetic homogenisation with loss of local adaptations in natural populations^33,34^. This risk is especially severe for salmonids, since local populations often have developed considerable genetic differences that have sometimes been evidenced to be adaptive^34–37^. Since many local salmonid populations are already struggling due to overfishing, habitat loss and/or degradation and climate change, they are particularly vulnerable to the effects of genetic introgression from non-native strains. However, in cases where wild resident brown trout populations are subjected to interbreeding with hatchery-reared fish, hybridization could possibly induce the migratory life-history strategy in the receiving population with the potential cost of losing other adaptive qualities.

**Figure 1:**
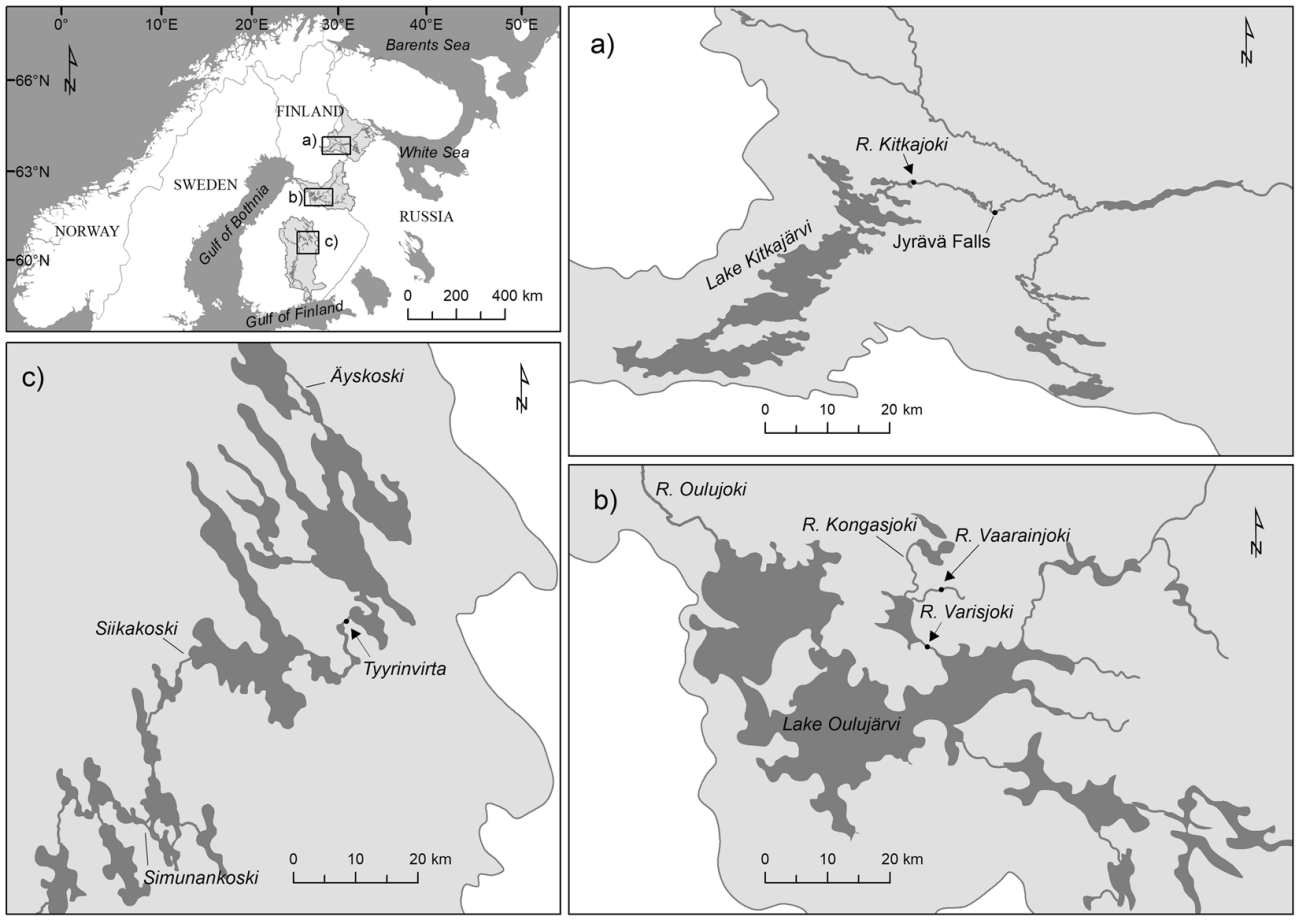
Map illustrating the geographical origins of the study strains. (**a**) Lake Kitkajärvi (KIT), (**b**) River Oulujoki watercourse (OUV) and River Vaarainjoki (VAA), respectively, and (**c**) Rautalampi watercourse (RAU), that covers larger area. The arrows indicate the main locations where the wild parental fish have been caught.

In this study, we assessed how experimental hybridization of a hatchery-strain of migratory brown trout (*Salmo trutta*) females with males of a wild resident but local population (from the same river system) and with males from two geographically distinct migratory hatchery strains (Fig. 1) affects the early survival, growth and personality of F_1_ offspring. We reared both the control offspring (purebred) and hatchery × wild hybrid offspring in a half-sib setting that allowed the estimation of quantitative genetic parameters for personality traits for the sub-set of fish in these two groups (Table 1). We predicted that crossing with wild males would decrease the boldness of the offspring, whereas crossing with geographically distinct males should increase variance among offspring and potentially cause outbreeding depression detectable as increased mortality and retarded growth^33,38^. We predicted that a long history of the strain in hatchery conditions (represented by River Oulujoki watercourse and Rautalampi watercourse strains) should result in increased boldness and survival of the juveniles in hatchery conditions.

**Table 1:** The F_1_ groups and their parents in the study.

F_1_ groups	♀ (n = 5)	♂ (n = 5)
OUV (purebred)	River Oulujoki (migratory strain)	River Oulujoki (migratory strain)
VAA (hybrid)	River Oulujoki (migratory strain)	River Vaarainjoki (wild resident strain)
KIT (hybrid)	River Oulujoki (migratory strain)	River Kitkajoki (migratory strain)
RAU (hybrid)	River Oulujoki (migratory strain)	Rautalampi watercourse (migratory strain)

## Results

### Mortality and growth

**Figure 2:**
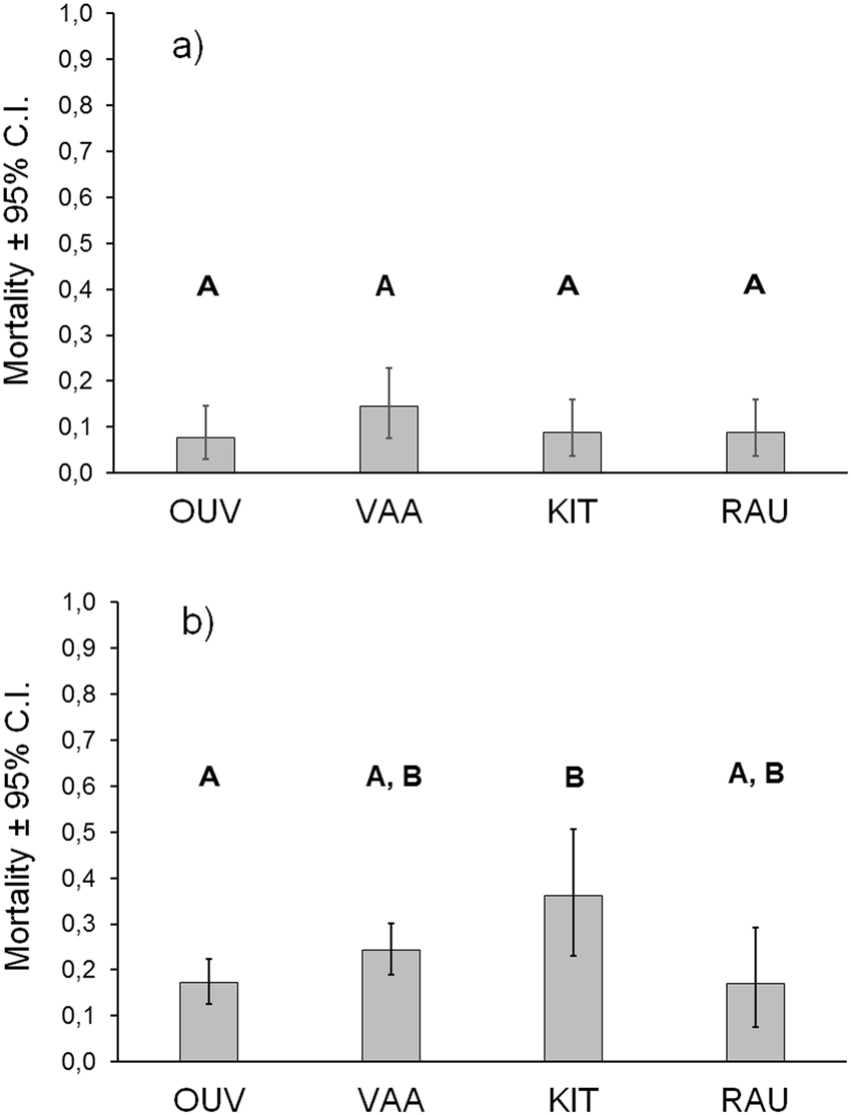
Least square mean mortalities (±their 95% confidence intervals) of the study groups in (**a**) incubation tubes (eggs and fry) and (**b**) rearing tanks (fingerlings). Different letters above the bars indicate a statistically significant pairwise difference between the groups. The values represent the actual (back-transformed) proportions.

The mean mortalities of the crossing groups were close to statistically significant difference during the egg-alevin period (linear mixed effects model; *F*_3,16_ = 2.88, *p* = 0.069; Fig. 2a). The VAA group had then somewhat higher mortality than the other groups. During on-growing, the differences of mortality means between the male strains were statistically significant (*F*_3,56_ = 3.04, *p* = 0.036; Fig. 2b). The KIT crossing group had the highest mortality during on-growing, and this group also differed significantly from the OUV group in pairwise comparisons (Fig. 2b).

After correcting the day of measurement for the first body length data, the initial differences of means between the crossing groups appeared to be non-significant (*F*_3,11_ = 3.00_,_ *p* = 0.077; linear regression for day effect, *F*_1,3.33_ = 14.76_,_ *p* = 0.026; Table 2). Instead, the body length means differed statistically significantly between the crossing groups both in the second (*F*_3, 57.3_ = 4.19_,_ *p* = 0.010; day effect, *F*_1,200_ = 203.05, *p* < 0.001; Table 2) and third measurement (*F*_3,50.7_ = 2.88_,_ *p* = 0.045; Table 2). At both latter times, the KIT group had the smallest body length mean, though in pairwise comparisons the only significant differences were found between KIT and OUV (second measurement) and between KIT and OUV or VAA (third measurement). At the final measurement time, the coefficients of variation in raw body lengths (CV %) were 9.6%, 11.6%, 10.6% and 8.8% for OUV, VAA, KIT and RAU, respectively.

**Table 2:** Mean total lengths (mm) of the fish from the different strains at the three measurement occasions (I: fry stage 25^th^ May–1^st^ June, II: fingerlings 27^th^ June–20^th^ July and III: one-summer old parr in 3^th^–4^th^ September).

Measurement	OUV Mean	95% C.I.	VAA Mean	95% C.I.	KIT Mean	95% C.I.	RAU Mean	95% C.I.
I	29.26	28.56–29.96	29.67	28.97–30.37	29.51	28.81–30.20	29.53	28.83–30.23
II	49.01	48.02–50.00	47.71	46.72–48.71	44.12*	41.52–46.72	47.82	45.43–50.21
III	78.08	76.71–79.44	77.80	76.36–79.24	73.10**	70.02–76.19	77.42	74.41–80.43

*Statistically significantly different from the OUV group. **Statistically significantly different from the OUV and VAA groups.

### Behavioral data

PCA yielded two principal components (PCs) with eigenvalue higher than one, explaining 80% of the total variation in the measured variables, that were all repeatable also themselves (Table 3). PCA without fish that did not come out from the start box yielded very similar results with at maximum 16.8% difference in absolute value loadings exceeding 0.5, suggesting that the extracted correlation structure was free of potential biases caused by the maximum values. PC1 explained 56.3% of total variation in observed behaviors. PC1 was interpreted, by inspecting the correlations of the different variables, to reflect boldness/shyness and exploration: this principal component consisted of latency to leave the starting compartment and reach the lines on the arena and the mirror at the other end of the arena (Table 3). PC1 (hereafter “exploratory tendency”) was also associated with a low number of visits to the mirror during the trial (Table 3). Therefore, the individual values of PC1 describe boldness and exploration in opposite direction, i.e. there is a negative correlation between PC1 and boldness/exploration (a high value depicting a shy and non-explorative individual). The second behavioral component (PC2, hereafter “freezing”), explaining 23.4% of the observed behavior, involved the number of times the fish were performing freezing behavior and the total time spent freezing during the trial (Table 3). Freezing-related original variables were weakly positively correlated with the latency times to reach different points (Pearson’s *r* = 0.086–0.136, *n* = 600, *p* = 0.001–0.036) indicating that the late emergence from the start box did not automatically reduce chances for freezing. Principal components, by definition, do not correlate with each other, and PCA removed the original negative correlation between boldness and freezing.

**Table 3:** Principal components extracted from PCA and behavioral variables included in the analysis (Varimax-rotated component matrix).

Variable	PC1	PC2	*r* (ICC)	*p*	# Max or min values
Ln (Total time spent in the start box)	0.869	0.035	0.141	0.007	38 (max)
Ln (Time to exit from the start box)	0.905	0.045	**0.102**	0.039	38 (max)
Ln (Time to reach the 1^st^ line)	0.962	0.067	0.199	<0.001	46 (max)
Ln (Time to reach the 2^nd^ line)	0.959	0.076	0.179	<0.001	61 (max)
Ln (Time to reach the mirror)	0.891	0.103	0.149	0.005	130 (max)
Ln (Times visited the mirror + 1)	−0.535	−0.419	0.396	<0.001	130 (min)
Ln (Number of times freezing + 1)	0.023	0.902	0.440	<0.001	92 (min)
Ln (Total freezing time + 1)	0.062	0.928	0.380	<0.001	92 (min)
Eigenvalue	4.639	1.742			
% of variance	57.945	21.770			

Repeatability (*r*) and significance (*p*) of the repeatability estimate is presented along with the number of maximum or minimum values for each variable (600 tests in total).

### Personality differences and heritability

Both PCs were repeatable among individual fish when the confounding fixed effects were corrected for in the genetic models (*r* = 0.199 ± 0.064 SE, and *r* = 0.418 ± 0.053 SE, for PC1 (exploratory tendency) and PC2 (freezing), respectively). The PC1 was heritable at a low level (*h*^2^ = 0.100 ± 0.065 SE). The permanent environment effect on PC1 was of the same magnitude as heritability (*p*^2^ = 0.098 ± 0.074). Heritability estimate for PC2 was even lower and associated with relatively high standard error (0.064 ± 0.060 SE). Instead, the permanent environmental effects on PC2, including non-additive genetic effects, were moderate (*p*^2^ = 0.354 ± 0.071). The common environmental effect due to separate rearing tanks between full-sib families was negligible for both PCs, and this additional variance term was consequently excluded from the genetic models (likelihood ratio test, PC1: *p* = 0.394; PC2: *p* = 0.500).

**Figure 3:**
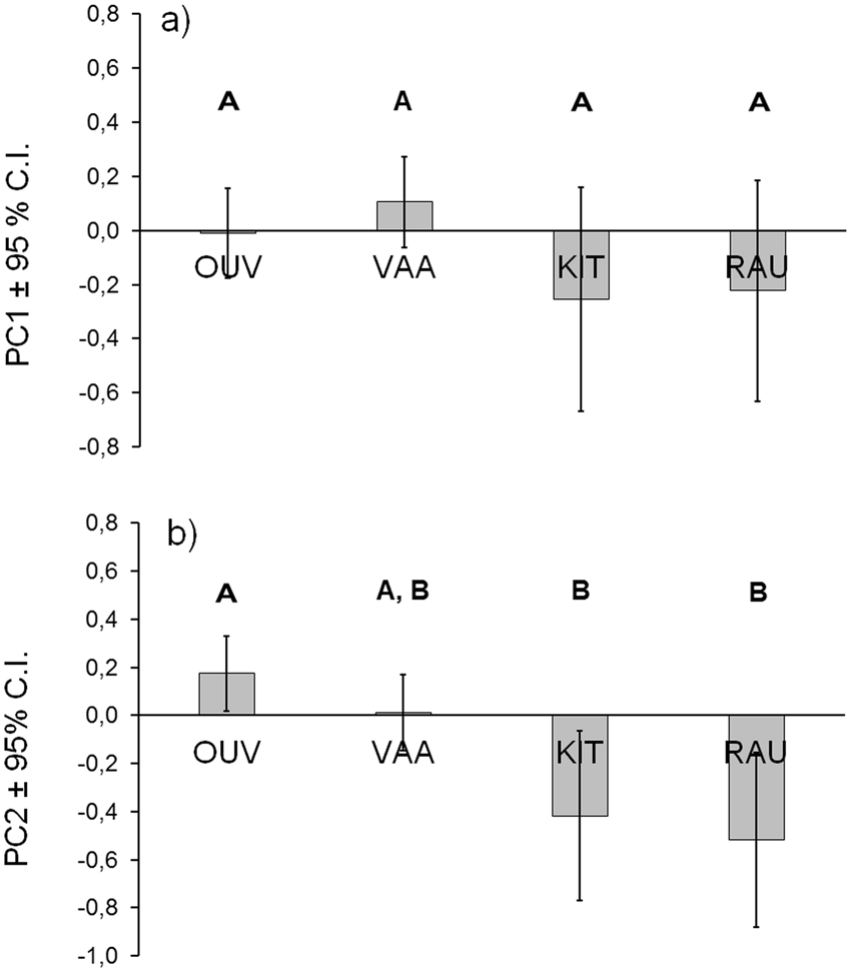
Estimated marginal mean values among the groups for the two principal components (**a**) for the PC1 (exploratory tendency) and (**b**) for the PC2 (freezing) describing the two personality axes. Different letters above the bars indicate a statistically significant pairwise difference between the groups.

From variables originally included in mixed models for PC1 (exploratory tendency), the length of fish and time of trial were removed, since their effect on PC1 was weak. The effect of the male strain was not statistically significant (*F*_3, 72.21_ = 1.093, *p* = 0.358) (Fig. 3a). The fish became less eager to start moving fast in the second trial (repetition: *F*_1, 525.43_ = 24.764, *p* < 0.001), and high water temperature (*F*_1,548.14_ = 4.984, *p* = 0.026) statistically significantly decreased the value of the PC1 (increased exploration). Inclusion of the non-significant effect of test day number (*F*_1,554.93_ = 2.19, *p* = 0.140) did not change the result of non-significant strain effect.

From the original variables in the mixed model for PC2 (freezing), water temperature, repetition and recovery time were left out from the final analysis, since their effect on PC2 was relatively low. The effect of male strain was statistically significant (*F*_3,59.31_ = 6.050, *p* = 0.001) (Fig. 3b). Fish length (*F*_1,560.44_ = 31.202, *p* < 0.001) increased freezing as well as late time of the day during the trial (*F*_1,556.41_ = 13.354, *p* < 0.001). In pairwise comparisons, two main groups were formed: OUV and VAA grouped together but only OUV differed statistically significantly from KIT and RAU that did not differ from each other (Fig. 3b). Inclusion of the non-significant effect of test day number (*F*_1,588.53_ = 1.490, *p* = 0.223) did not change the result of significant strain effect.

For both PCs, a statistically highly significant difference in additive genetic value means were found between the OUV and VAA groups (PC1: *F*_1,248_ = 13.12, *p* < 0.001; PC2: *F*_1,248_ = 18.19, *p* < 0.001). The VAA group had a larger genetic mean than OUV in PC1 (mean ± SD = 0.039 ± 0.156 (range −0.283 to 0.354) in VAA, and −0.039 ± 0.185 (range −0.493 to 0.314) in OUV), suggesting that the hybrid group was at additive genetic level more shy and less explorative than the purebred strain. Instead, the purebred OUV strain had higher mean of additive genetic values in PC2, compared to the hybrid VAA group, although the genetic variance in this personality component was probably minor (mean ± SD = −0.029 ± 0.094 (range −0.291 to 0.168) in VAA and 0.029 ± 0.117 (range −0.249 to 0.239) in OUV). Thus, the purebred OUV strain may have a weaker additive genetic tendency for freezing than their hybrid conspecifics.

The OUV and hybrid VAA group did not differ in their means of individual permanent environment effect values (PC1: *F*_1,248_ = 0.52, *p* = 0.470; PC2: *F*_1,248_ = 0.86, *p* = 0.355), which suggests that the non-additive (genetic) effects on the studied personality components were similar between the purebred and hybrid strains.

## Discussion

We found that paternal strain explained phenotypic differences among hybrid brown trout juveniles in mortality, growth and a personality component illustrating freezing behavior (PC2). Freezing behavior is often interpreted as indicative of high sensitivity to stress and thus expectedly selected against in hatchery conditions^39^. Moreover, the purebred migratory strain (OUV) possessed a stronger additive genetic tendency for boldness and explorative behavior, and weaker genetic tendency for freezing behavior than the resident wild × hatchery hybrid group (VAA). This provides first experimental evidence for the genetically mediated behavioral consequences that stockings of fish from non-native strains can cause. These personality axes are known to have a role in ecological interactions and setting the vulnerability of individual fish to fishing^40,41^. Thus, these results indicate that ecologically relevant genetic differences, including those affecting life-history strategies^42^, can occur among geographically very close populations (in this case within 5 km range), and add to the evidence of risks arising from the mixing of different salmonid strains by stockings^34,43^. However, as earlier inbreeding depression in small fragmented populations is a serious threat^44^, and as the research on salmonids concerning inbreeding depression versus outbreeding depression has documented fairly mixed and unpredictable effects^16,43^, the relative costs and benefits of mixing endangered populations should be evaluated on case-by-case basis^41,43,44^. In this case, the OUV population was founded using fish from multiple origins, which has provided the presence of relatively large genetic diversity in this broodstock despite the limited population size^42^.

Due to their longer history in hatchery conditions, both OUV and hybrid RAU group were expected to have a low early mortality in hatchery conditions. Due to relatively low early mortality in all F_1_ groups, however, no differences were found during the egg-alevin stage. Instead, mortality during on-growing differed between the groups as the KIT crossing group suffered from the highest mortality. The higher mortality together with approximately 6% slower growth in KIT fry, relative to the three other F_1_ groups, seems to reflect potential outbreeding depression between geographically distant River Oulujoki watercourse and Lake Kitkajärvi strains. In general, outbreeding effects can vary between taxa, characters being measured, level of divergence between hybridizing populations, mating history, environmental conditions and the potential for inbreeding and outbreeding effects to be occurring simultaneously^44^. Brown trout populations show very fine-scaled genomic differences indicating that geographical proximity does not necessarily reveal genetic relatedness^45^. Moreover, the present results could partially have been confounded by geographical distance and by different sample sizes for the third length measurement round. Therefore, further detailed interbreeding studies using controlled mating set-ups are needed to understand the interplay between local adaptation and high population level genetic variation.

The assessed behaviors reflect boldness and stress-related personality axes under experimental conditions and partially support previous results on similar repeatable personality components (PCs) and stress coping styles in the brown trout^39^. In line with two previous studies in brown trout juveniles, the boldness-reflecting behavioral traits were individually consistent and showed heritability^39,40^. In the present study, a common genetic variance was estimated for the migratory purebred (OUV) and half-resident hybrid VAA group, because the number of families (and tested fish within families) was fairly low to estimate genetic parameters separately for each group. The standard errors relative to the genetic parameter estimates were nevertheless large, and the variance estimates must be treated with caution. The recent finding in Finnish brown trout (purebred RAU line) of freezing-related behaviors being more heritable than boldness itself^39^ also falls within the statistical confidence limits and suggests altogether that both boldness and freezing show low heritability of 0.05 to 0.1 in brown trout. The additive genetic value mean in PC1 was higher for the half-resident hybrid VAA group than for the migratory purebred OUV group, indicating that the hybrid fish may exhibit a weaker (additive) genetic tendency for boldness and non-explorative behavior. Correspondingly, based on the genetic value means in PC2, the hybrids may have a stronger genetic tendency for freezing than the purebred hatchery strain. This finding gives partial support to previous behavioral studies where farmed-wild hybrids have been found to differ phenotypically from their wild parental stock in boldness and aggressiveness^5,13–15^.

No statistically significant differences in phenotypic PC1 values (indicating explorative tendency and boldness) were found between the F_1_ groups. Instead, PC1 values were explained more by the test number (first or second) and water temperature that was confounded with the testing date and water oxygen concentration. However, the additive genetic estimate of boldness in the hatchery line was higher than for the wild x hatchery crossed VAA group which suggests that evolutionarily relevant differences may not always manifest themselves as phenotypic differences in experimental conditions and in simple models excluding family effects. Even a few generations in hatchery conditions have been shown to induce significant genetic changes in a multitude of traits^24–26^. Personality traits like boldness are often associated with ecologically important processes such as increased risk by predation and parasitism^41^, thus suggesting that the observed differences in laboratory setting may have significant survival implications.

Considering PC2, both the purebred OUV and half-resident hybrid VAA groups displayed higher tendency for freezing than the hybrid KIT and RAU groups, possibly indicating higher sensitivity to stress in the former groups. Since PC2 involved low additive genetic variation, the results can be caused by the non-additive genetic factors (i.e., permanent environment effects were moderate for PC2). Since behavioral tests were performed later for KIT and RAU hybrid groups than for purebred OUV and hybrid VAA groups, the effect of date could not be entirely separated from the effect of sire’s origin despite the testing date did not appear statistically significant in formal testing. Neither water temperature nor oxygen concentration had a statistically significant effect on PC2, so it is unlikely that statistically significant differences between F_1_ groups would be entirely due to environmental changes during the experiment period. It is also unlikely that the results would have been affected by the growth of the fish during experiment period, since the positive effect of length on PC2 was controlled for in the test (larger fry had higher PC2 -scores than smaller ones). Given that both the KIT and RAU groups had a long hatchery breeding history, it is a valid suggestion that the lower freezing tendency in these hybrid groups resulted from past adaptation to stressful hatchery conditions. Nevertheless, this result should be interpreted under the assumption that developmental stage does not associate with nonlinear changes in the sensitivity to stress^46^. In general, the fairly short duration of the behavioral trials in our study was considered long enough to provide repeatable and credible results, as in our earlier work virtually all the study fish exited the starting compartment^39^. Also in the present study, the majority (264/300) of the fish exited the starting compartment during the experimental time in the both trials. Only two fish stayed in the starting compartment in the both trials.

To conclude, our results on brown trout juveniles add to the evidence that personality traits involve heritable variation in fish and significant differences can be found between populations from very close geographical locations. Personality differences between hybrid and purebred strain trout appeared to occur at genetic level, which calls for caution in stockings involving the risk of hybridization. The present findings also warn about the occurrence of outbreeding depression in early mortality and growth between genetically and geographically distinct strains.

## Methods

### Study fish

Handling and rearing of fish were conducted in accordance with the National Animal Experiment Board’s approval (ESAVI/2458/04.10.03/2011). All animal experimentation reported meets the ABS/ASAB guidelines for ethical treatment of animals and comply with the current Finnish legislation. The parents of this study fish were obtained from the broodstocks maintained by the Finnish Game and Fisheries Research Institute (currently the Natural Resources Institute Finland), and represented populations that show economic or scientific importance in Finland. All females originated from the River Oulujoki watercourse broodstock (3^rd^ or 4^th^ hatchery generation) that had originally been founded using wild brown trout from Rivers Varisjoki and Kongasjoki, both discharging to the Lake Oulujärvi (Fig. 1). For the veterinary reasons, we were able to use females only from this population. The females were mated with males from the same strain (also representing 3^rd^ or 4^th^ hatchery generation fish) as well as with males from three other brown trout strains, thus resulting in one control (purebred) F_1_ group and three hybrid F_1_ groups (Table 1). The sires of the three hybrid groups originated from the River Vaarainjoki (located next to River Kongasjoki and upstream from River Varisjoki with one lake between, wild-caught individuals, Fig. 1), the Lake Kitkajärvi (1^st^ hatchery generation, strain above the Jyrävä waterfall) and the Rautalampi watercourse (5^th^ or 6^th^ hatchery generation). The Rautalampi hatchery strain represents a collection of several origins of fish (Äyskoski, Tyyrinvirta, Siikakoski and Simunankoski, Fig. 1) and had the longest history of captive breeding in a hatchery. Brown trout from River Vaarainjoki and River Oulujoki watercourse broodstock are moderately genetically differentiated (F_ST_ = 0.109 based on 4876 SNP loci, Prokkola *et al*. submitted MS 2018). Genetic distance (F_ST_) between River Oulujoki watercourse broodstock and Rautalampi hatchery strain is at the level of 0.073 (M.-L. Koljonen and J. Koskenniemi, unpublished data 2016 based on 16 microsatellite markers). Lake Kitkajärvi strain has not yet been compared to the other included strains. Apart from the resident River Vaarainjoki strain, the other strains were classified migratory. This classification was based on original status of the stocks taken to hatcheries, indirect genetic evidence (large heterogeneity indicates migratory status)^43^ and experimental evidence between OUV and VAA populations (authors’ unpublished data). We use the following abbreviations to identify the F_1_ groups: OUV (River Oulujoki ♂ × River Oulujoki ♀), VAA (River Vaarainjoki ♂ × River Oulujoki ♀), KIT (Lake Kitkajärvi ♂ × River Oulujoki ♀) and RAU (Rautalampi watercourse ♂ × River Oulujoki ♀).

Five females from the River Oulujoki watercourse strain and five males from each of the four strains (20 males in total) were used for fertilizations that were carried out in October 12^th^ in 2011, resulting in 100 female-male combinations (i.e. 25 half-sib families per group). The fertilizations took place at the Kainuu Fisheries Research Station (www.kfrs.fi), where all the F_1_ offspring were raised in the same hatchery conditions. Each fertilization combination was divided into three equal replicates (300 incubation units in total, approx. 100 eggs each). The incubation units were open plastic tubes with polystyrene floats and a mesh bottom (100 mm in length and diameter). The incubators were divided into six flow-through tanks (3 m long, 50 incubators per tank) so that OUV and KIT families were always in the same three tanks and KIT and RAU families together in other three tanks. After the first three days, the egg count for the fertilized eggs per each unit was 89 ± 0.85 (mean ± S.D) eggs per unit. The water used in the rearing tanks came from the adjacent Lake Kivesjärvi, and the variations of temperature and oxygen levels during the study followed those in natural conditions.

After the first three days, the incubation units were moved to circular 3.2 m^2^ tanks (water volume 800 l), where they floated vertically in. Eggs and alevins were incubated in these tanks in three replicates until May 2012. All eggs hatched by 20^th^ of March 2012. On 21^st^−23^th^ May in 2012, a total sample of 6300 start-feeding fry was transported to 60 separate rearing tanks (surface area 0.4 m^2^; water volume during the first two weeks 80 l, then 160 l) for on-growing until the end of the experiment. The feeding of the fry was also started at this time. The fish were fed *ad libitum* by automatic belt feeders with commercial dry salmonid food (Biomar INICIO plus G; 0.4–1.1 mm). The offspring of males from the OUV and VAA strains were placed in 50 tanks in full-sib families consisting of 105 individuals/family. The families were formed by selecting the same number of individuals from each of the three egg incubation replicates for further rearing, if possible (35 fish/repetition in all but two cases). For logistic constrains, the half-sib families of males from KIT and RAU strains were placed together in 10 tanks so that in each tank there were 105 individuals that shared the same male parent but not the same female parent. These half-sib families were also formed by taking the same number of individuals from each female-male fertilization replicate (7 fish repetition^−1^ in all cases). As a result of these combinations, 25 tanks were formed for both OUV and VAA full-sib families, and 5 tanks for both KIT and RAU half-sib families. Behavioral experiments were performed for five individuals from each of these 60 rearing tanks (for 300 individuals in total, see below).

### Mortality of the offspring

The mortality of the F_1_ offspring was monitored every few days during the egg incubation and hatching period (from fertilization on 12^th^ October 2011 until 21^th^ May 2012) and then daily during the period of on-growing (from 24^th^ May 2012 until 6^th^ September 2012). Both dead eggs and fry were counted and removed.

### Body length measurements

The first body length measurements were performed between 25^th^ May–1^st^ June 2012. After transporting 6300 start-feeding fry into rearing tanks for on-growing and behavioral experiments, 1180 of the fry remaining in the incubation tubes were measured for total body length (29.5 ± 1.2 mm, mean ± S.D). Four individuals from each of the 300 incubation tubes were haphazardly selected for the measurement, except for the six cases where there were less than four “surplus” individuals remaining after the transport (in these cases 0–3 individuals were measured). Since all the incubation tubes having less than four surplus individuals belonged to VAA test group, slightly smaller sample sizes for VAA test group was used (280 measured individuals from VAA group and 300 from each of the other three test groups). The fish sizes were measured in a rotating order, so that four individuals from 25 OUV tubes were measured first, then the same number from KIT tubes, RAU tubes and finally from VAA tubes.

The second body length measurements took place approximately one month later, between 27^th^ June and 20^th^ July 2012, when 300 individuals (48.0 ± 5.7 mm, mean ± S.D), five individuals/tank, were measured as a part of the behavioral assays. Haphazard netting of the study fish according to a randomized order of the rearing tanks (families) was used to select the individuals for behavioral trials and subsequent measurements. However, the order of the rearing tanks was randomized in a way that both OUV and VAA individuals were tested and measured first (27^th^ June to 19^th^ July), whereas individuals from KIT and RAU hybrid populations were tested and measured during the last four days of the experiments (between 17^th^ and 20^th^ July 2012). OUV and VAA groups were prioritized to secure the quantitative genetic parameter estimations in the case of any disease epidemics. The measurement order thus resulted in greater body length and weight values for KIT and RAU crossing groups, and this bias was accounted for in the analyses and interpretation of the results.

Third measurement period took place in 3^rd^–4^th^ September in 2012. At this time, 1113 individuals remaining in the rearing tanks were measured for their body lengths (77.4 ± 8.2 mm, mean ± S.D). This measurement group consisted of 450 offspring of OUV and VAA males, 108 offspring of KIT males and 103 offspring of RAU males.

### Quantification of behavioral traits and personality

The behavioral trials quantifying individuals’ boldness and exploration tendency were performed between 27^th^ June and 20^th^ July 2012. Five haphazardly dipnetted individuals from each of the 60 full-sib or half-sib families were included in the experiment (300 individuals in total: 125 individuals from OUV and VAA groups and 25 individuals from KIT and RAU groups). The study fish were deprived of food for approximately 36 hours before the experiment individually in small acclimation tanks (140 × 120 mm, water depth approx. 50 mm).

In the personality assay, the study fish were placed one at a time in a specially made emergence test tank (see details in^39^), that consisted of a darker-walled starting compartment, i.e. box, (separated from the rest of the tank by a door that could be lifted from a distance by pulling a line) and a larger, lighter-walled test arena (with uniform light gray floor). On the bottom of the test arena, two drawn lines allowed us to evaluate the time that it took for the study fish to swim further into the arena (to cross the first and the second line). To measure the boldness and exploration tendency of the study fish, the test tank included two rocks for shelter and a mirror covering the end wall of the arena. At the beginning of the trial, the study fish were placed in the starting compartment, where they were allowed to acclimate to the circumstances for three minutes. After the acclimation period, the door of the starting compartment was lifted, allowing the fish to swim into the test arena. We used software assisted timing (custom software by A.V.) to record the time it took for the fish to activate (move for the first time in the starting compartment), leave the starting compartment, swim over the lines in the arena and touch the mirror at the other end. We also recorded if the fish swam back to the starting compartment, touched the mirror for more than one time or exhibited freezing behavior (stayed motionless for more than one second, as indication of fear or stress^39^. The duration of the experiment was eight minutes from the moment the door of the starting compartment was lifted.

As expected, not all study fish performed all of the behaviors described above during the test period. To prevent any bias in the data, it was important to include these individuals in statistical analysis. Therefore, when a value was missing for any behavioral variable, a maximum value was used (the duration of a trial, 8 min). For example, if an individual never activated during the trial, it was given the maximum value (8 min) for activation and all possible further behaviors (entering the test arena, crossing the first and second line in the arena and touching the mirror at the other end). Maximum values were used because they represented the actual behavior of more passive study individuals better than not giving them any values, in which case the study group would have seemed, on average, more active and/or bold than in reality.

The behavioral trial was performed twice for each tested individual to enable the evaluation of short term repeatability of behavior^39^. Both trials were always performed on the same day, with at least three hours in between the trial times to recover from possible stress caused by the first trial (recovery time 258.01 ± 38.79 min, mean ± S.D). After the trials the study fish were euthanatized using an overdose of anesthetic (clove oil, 500 mg l^−1^) and their lengths and weights were measured.

### Statistical analysis

#### Mortality

Mortality was analyzed separately for the egg incubation-alevin period and for the period of on-growing (from start-feeding onwards). Since the number of fertilized eggs per family varied slightly in the beginning of the experiment (mean ± S.D. = 89.3 ± 14.8, range 40–143), arcsine square root transformed proportions of dead eggs were used in the analysis. The number of fish in each half-sib family was equalized when the alevins were transported to the rearing tanks (105 individuals per tank). However, due to a block in a faucet, 58 individuals died in one of the rearing tanks. To include this tank in the data, arcsine square-root transformed proportions of mortality were also used in the analysis of this period (deaths caused by the block were not included in the mortality data). The mortalities during both periods were analyzed using a linear mixed effect model using restricted maximum likelihood estimation (REML) in SAS 9.4. software (SAS Inst. Inc., Cary, NC). The significance of different random effects in the models (e.g. incubation tank or male identity, nested within male strain, or interaction between female and male strain) was separately tested by comparing the goodness of fit of the alternative models either containing or missing the effect (likelihood ratio test with one degree of freedom)^47^. Further, the appropriate error structures were chosen for the models based on the values of Akaike’s Information Criteria (AIC). Insignificant (co)variances were excluded from the final models. For the first (egg-alevin) period, the model was:

$${y}_{ijkl}={\mu }+{\rm{male}}\,{{\rm{strain}}}_{j}+{{\rm{female}}}_{k}+{{\rm{male}}}_{l(j)}+{e}_{ijkl},$$

where *y*_*ijkl*_ is the arcsin square root transformed mortality within an incubator *i*, *µ* is the model intercept (overall population mean), male strain_*j*_ is the fixed effect of male strain (*j* = 1–4), female_*k*_ is the random effect of female parent (*k* = 1–5), male_*l*(*j*)_ is the random effect of male parent (*l* = 1–20), nested within male strain, and *e*_*ijkl*_ is the random error term.

For the second (on-growing) period the model was:

$${y}_{ij}={\mu }+{\rm{male}}\,{{\rm{strain}}}_{j}+{e}_{ij}$$

where *y*_*ijm*_ is the arcsin square root transformed mortality within a rearing tank *i* (*i* = 1–60 tanks). Tukey-Kramer -type post hoc tests were used to identify pairwise differences among the four male strains.

#### Body length measurements

Body length differences among the male strains were tested separately at the three different measurement periods (period 1: 1180 fry measured between 25^th^ May–1^st^ June 2012, period 2: 300 fingerlings measured between 27^th^ June–20^th^ July 2012 and period 3: 1113 individuals measured between 3^th^–4^th^ September 2012). For the first period, the linear mixed effect model was:

$${y}_{ijklm}={\mu }+{\rm{male}}\,{{\rm{strain}}}_{j}+{{\rm{day}}}_{m}+{{\rm{female}}}_{k}+{{\rm{male}}}_{l(j)}+{e}_{ijklm},$$

where *y*_*ijklm*_ is the body length of an individual *i*. Residual covariances among individual fish were estimated for each incubation tank separately. Further, because the first measurement period lasted for 9 days the day of measurement (calculated since the beginning of the measurement period in question) was included in the model as a fixed covariate.

For the second and third periods, the linear mixed model was of form:

$${y}_{ijklm}={\mu }+{\rm{male}}\,{{\rm{strain}}}_{j}+{{\rm{day}}}_{m}+{{\rm{tank}}}_{n(j)}+{e}_{ijklm},$$

where tank_*n*(*j*)_ is the random rearing tank effect (*n* = 1–60), nested within male strain. It is noteworthy here that KIT and RAU fish were kept in paternal half-sib tanks, and consequently in these two groups the tank effect on fish growth may be partially confounded with maternal identity effect. The date of measurement (*m* = 1–23 days) was included as a fixed covariate for the model of the second length data only. Separate residual variances were estimated for each male strain. Tukey-Kramer pairwise comparisons were used to find which male strains differed from one another.

#### Behavioral data

The assumption on normal distribution of residuals was tested using one-sample Kolmogorov-Smirnov test. Since the normality of some variables was improved by logarithm transformation, Ln-transformation was used for all variables (Ln(X + 1) was used for number of times mirror touched, number of times frozen and freezing time). The repeatability of individual behavioral variables was analyzed using the Interclass Correlation Coefficient (ICC)^48^ prior to inclusion in PCA, since analyzing the heritability of a non-repeatable behavior would not be reasonable^49^.

Principal component analysis (PCA; IBM SPSS Statistics) with varimax rotation was used to combine multiple behavioral variables into uncorrelated principal components (PC), as this approach has been adopted in recent personality studies in fish^39^, which allows us to compare the results with earlier studies. The variables included in PCA were (1) entering the arena, (2) crossing the first and (3) second line in the arena, (4) touching the mirror (for the first time during trial), (5) number of times the mirror was touched during the trial, (6) time spent in the starting compartment, (7) number of times the individual showed freezing behavior and (8) total time spend freezing during the trial. Behavioral data from all groups was included in the same principal component analysis.

The genetic parameters for the two obtained PCs were analyzed using REML estimation in ASReml 3.0 software^50^. Because the identity of both male and female parents were only known for OUV (control) and VAA crossing groups, the data from only these two groups were used for the genetic models. Due to a relatively low number of families (and low number of tested offspring per family) within the groups (125 fish per strain), the genetic models were run for a combined data including both groups together. Estimation of common genetic variances for the two crossing groups is justified as these groups are not genetically independent, separate populations but share the same mothers. The variance components for each PC were estimated using a repeated measures animal model, which can be written in matrix notation as:

1 $$y={\bf{X}}{\bf{b}}+{{\bf{Z}}}_{a}{\bf{a}}+{{\bf{Z}}}_{p}{\bf{p}}+{\bf{e}}$$

where *y* is the vector of individual PC scores, **b** is the vector of fixed effects, **a** is the vector of random additive genetic effects, **p** is the vector of random permanent environment effects and **e** is the vector of random residual effects. The **X** is the design matrix associated with **b**, and **Z**_***a***_ and **Z**_***b***_ are incidence matrices assigning observations to the levels of additive genetic effects and permanent environment effects (i.e., non-additive contributions to fixed among-individual differences), respectively. Random variables **a**, **p** and **e** were assumed to be normally distributed. Specifically, ${\bf{a}} \sim N(0,\,{\bf{A}}{\sigma }_{a}^{2})$, where ${\sigma }_{a}^{2}$ is the additive genetic variance and ${\bf{A}}$ is the additive genetic relationship matrix derived from the parental generation; ${\bf{p}} \sim N(0,\,{\bf{I}}{\sigma }_{pe}^{2})$, where ${\sigma }_{pe}^{2}$ is the common environment variance; $e \sim N(0,\,{\bf{I}}{\sigma }_{e}^{2})$, where ${\sigma }_{e}^{2}$ is the residual variance and ${\bf{I}}$ is the identity matrix.

Further, the significance of an additional variance due to random rearing tank of individuals was also tested using the likelihood ratio test^47^.

Conditional Wald statistics was used to evaluate the significance of the fixed effects. Only the variables with significant contribution to the variation of behavioral PCs were included in the final models (*P* < 0.05). For both behavioral PCs, the confounding effects of water temperature and testing time (in minutes from 00:00) were fitted in the model as fixed covariates. For PC1 (exploratory tendency), the fixed effects also included the number of testing day (0–22) whereas for PC2 (freezing) fish body length was included.

The repeatability (*r*) of both behavioral PCs was calculated as:

2 $$r=\frac{{\sigma }_{a}^{2}+{\sigma }_{pe}^{2}}{{\sigma }_{a}^{2}+{\sigma }_{pe}^{2}+{\sigma }_{e}^{2}}$$

Correspondingly, heritability (*h*^2^) and permanent environment effect ratio (*p*^2^) were calculated for each PC as:

3 $${h}^{2}=\frac{{\sigma }_{a}^{2}}{{\sigma }_{a}^{2}+{\sigma }_{pe}^{2}+{\sigma }_{e}^{2}}\,{\rm{and}}\,{p}^{2}=\frac{{\sigma }_{pe}^{2}}{{\sigma }_{a}^{2}+{\sigma }_{pe}^{2}+{\sigma }_{e}^{2}},\,{\rm{respectively}}{\rm{.}}$$

Approximate standard errors were calculated for estimated variance components and variance ratios using ASReml.

We tested for differences in group means in individual additive genetic solutions (i.e., best linear unbiased predictions (BLUPs) of breeding values obtained from ASReml) for both PCs using a linear mixed model in SAS (group as a fixed effect). Similarly, the difference of means in individual permanent environment effect solutions was tested between OUV and VAA groups.

To analyze whether there were differences among the four F_1_ groups in the two behavioral PCs, linear mixed effect (LME) models were fitted to the data in SPSS 23.0.02 (IBM Corp, USA). Environmental variables that might have had an effect on the behaviors were included in the model. The variables included were water temperature and oxygen level (measured daily), repetition (1^st^ or 2^nd^ trial), size of the fish (measured as body length at the day of the behavioral trial), time of the trial (as minutes from 00:00 am), and recovery time between the trials (as minutes). Date was controlled by including strongly correlated water temperature as a covariate (Pearson’s *r* = 0.88, *p* = 0.01) and testing the day effect separately by adding it to the final model. Neither tank effect nor maternal effects could be independently included in the model. This was because each tank contained offspring from just one male parent (and from just one female parent in the case of OUV and VAA offspring). Since the offspring of both KIT and RAU sires were combined into half-sib families, the identity of the female parent was not known for these groups. Thus, the rearing tank identity, nested within male strain was included in the model as a random effect to control for the dependency arising from the common rearing environment. Bonferroni -type post hoc tests were used for pairwise comparisons of the four F_1_ groups. Model residuals were inspected for normality and found to satisfy the model assumptions.
